# Keystone Veterinary Medical Association

**Published:** 1897-04

**Authors:** W. L. Rhoads

**Affiliations:** Secretary


Secretary.

### KEYSTONE VETERINARY MEDICAL ASSOCIATION.

The February meeting was held the 9th inst. with the following members of the profession present: Drs. R. S. Huidekoper, H. D. Gill, H. D. Martein, W. W. Martin, J. D. Houldsworth, S. J. J. Harger, Otto G. Noack, H. P. Eves, J. R. Hart, W. Horace Hoskins, Charles Lintz, James T. McAnulty, T. B. Rayner, and W. L. Rhoads.

Rev. J. D. Diederick, of Flowertown, Pa., gave a very interesting talk on "The Silo Ensilage; Ensilage-fed Cattle, their Condition and Results." His talk showed him to be thoroughly conversant with his subject, he having made a practical study of this question for several years in its most minute detail, thus making the talk doubly pleasant and interesting to the practical veterinarian who is interested in cattle practice or has given the food question any study. He thought the white Southern corn the best variety, as the stalk is sweet and succulent as well as the ear. The better plan of planting is three or four feet apart and three or four grains to the running foot. This, if cut when the ears are in good condition for boiling, then shredded (as this is much better than the old method, which chopped the stalks and ears; the shredding not only puts the fodder itself in better condition, but has the corn more evenly distributed), and at this time properly housed, makes, if opened rightly (that is, by removing all the decaying top at first), a fine June food for the balance-ration of winter, and it should be used only as a balance-ration, for as a feed alone it soon causes the cattle to break down; but if used properly it is *par excellence*, taking the place of roots, etc., and is a better appetizer, keeping the flow of milk regular. At the conclusion of his much-appreciated talk Mr. Diederick was stormed with questions. These he readily answered, amply earning the hearty vote of thanks later extended to him by the Association.

The Association now adjourned its meeting and went in a body to the banquet-room in the Odd Fellows' Temple, where a luncheon was awaiting them, and if our figure-heads at Washington took half the interest in doing their duty toward Cuba that these veterinarians did in doing justice to the duty before them, Cuba Libre would be established within twenty-four hours.

After the invigorating influence of the Blue Points had begun to be felt, mixed, perhaps, with some of the *food-product* that has served to give one of our Western cities world-wide renown, then we heard how the Association had been useful in the home of one veterinarian, being for him a means of escape when probably a jack-pot was to be opened elsewhere; we then learned of the productiveness of farms in the northeastern section of Jersey. We learned also that while corn grew there very sparingly, the air seemed just suited to crows and dogs. We heard from Dr. Gill what progress science was making in the line of toxins. After his interesting talk we were entertained by Dr. Huidekoper telling us what he thought of toxins and what he knew of navicular disease. Those who know Huidekoper know he is always interesting, and this was certainly no exception to his general rule. In fact, everyone was called upon to say something, and each did his part, giving out some new ideas or brightening up some dark spots within the horizon of the veterinarian who is now striving to keep his profession on an honorable plane for the advent of better times.

The adjournment from this pleasant second session of the February meeting was now made on account of the distance some were from home.

W. L. RHOADS,
Secretary.

